# Supplementary material for: Rapid motor fluctuations reveal short-timescale neurophysiological biomarkers of Parkinson’s disease
Source: J Neural Eng. Author manuscript; Available in PMC 2021 May 22. (PMC8140652; doi:10.1088/1741-2552/abaca3)
Supplement: Supplementary Figures [file NIHMS1694256-supplement-Supplementary_Figures.pdf]

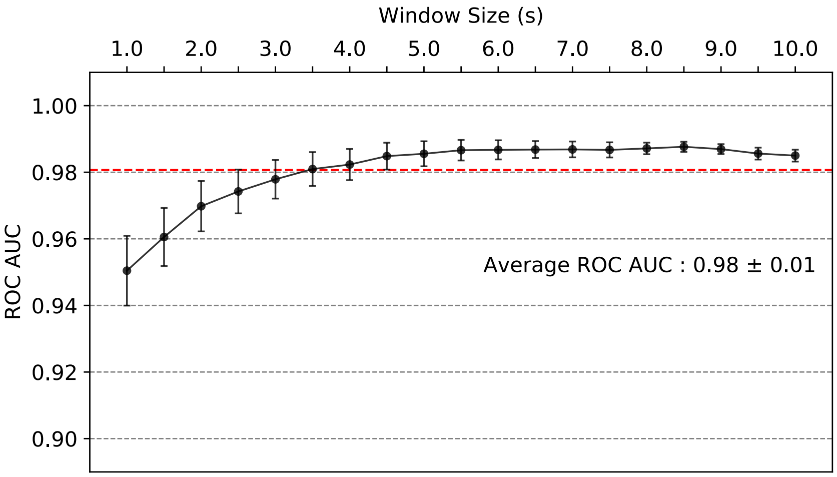

Supplementary Figure 1. *Classification of PD subjects vs. controls across timescales.* The multi-metric approach to behavioral assessment, yielding the MES, enabled good discrimination between control subject performance and PD subject performance at all tested timescales. Shown are the means ± standard errors for all subjects and sessions at each timescale.

A

θ/α	4.00–6.00 Hz
	5.00–7.00 Hz
	6.00–8.00 Hz
	7.00–9.00 Hz
	8.00–10.00 Hz
	9.00–11.00 Hz
	10.00–12.00 Hz
β	12.00–16.50 Hz
	14.25–18.75 Hz
	16.50–21.00 Hz
	18.75–23.25 Hz
	21.00–25.50 Hz
	23.25–27.75 Hz
	25.50–30.00 Hz
γ_{low}	30.00–37.50 Hz
	33.75–41.25 Hz
	37.50–45.00 Hz
	41.25–48.75 Hz
	45.00–52.50 Hz
	48.75–56.25 Hz
	52.50–60.00 Hz
γ_{mid}	60.00–70.00 Hz
	65.00–75.00 Hz
	70.00–80.00 Hz
	75.00–85.00 Hz
	80.00–90.00 Hz
	85.00–95.00 Hz
	90.00–100.00 Hz
γ_{high}	100.00–125.00 Hz
	112.50–137.50 Hz
	125.00–150.00 Hz
	137.50–162.50 Hz
	150.00–175.00 Hz
	162.50–187.50 Hz
	175.00–200.00 Hz
vhf	200.00–250.00 Hz
	225.00–275.00 Hz
	250.00–300.00 Hz
	275.00–325.00 Hz
	300.00–350.00 Hz
	325.00–375.00 Hz
	350.00–400.00 Hz

Supplementary Figure 2. *Frequency range definitions.* **A.** The 4–400 Hz range was divided into 6 "canonical" bands, which were each then subdivided into 7 sub-bands to serve as finer-grained features for MES decoding using neural activity.

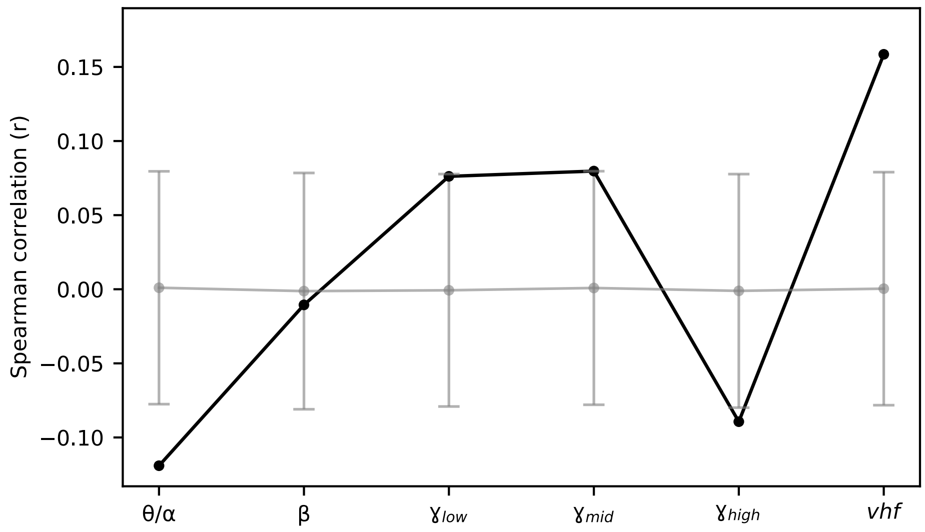

Supplementary Figure 3. *Relationship between peak spectral power and neural SVR weights.* For each canonical frequency band, spectral feature weights from the neural SVR for each recording were correlated with the peak spectral power normalized within-recording across-spectrum (n=161 recordings). No significant relationships were identified for any band (n=7 bands, Spearman's p-value > 0.05, corrected for multiple comparisons with q = 0.05). The black line depicts the empirical correlation, and the gray line shows the bootstrap null distribution with 10,000 samples (Error bars are standard deviations).

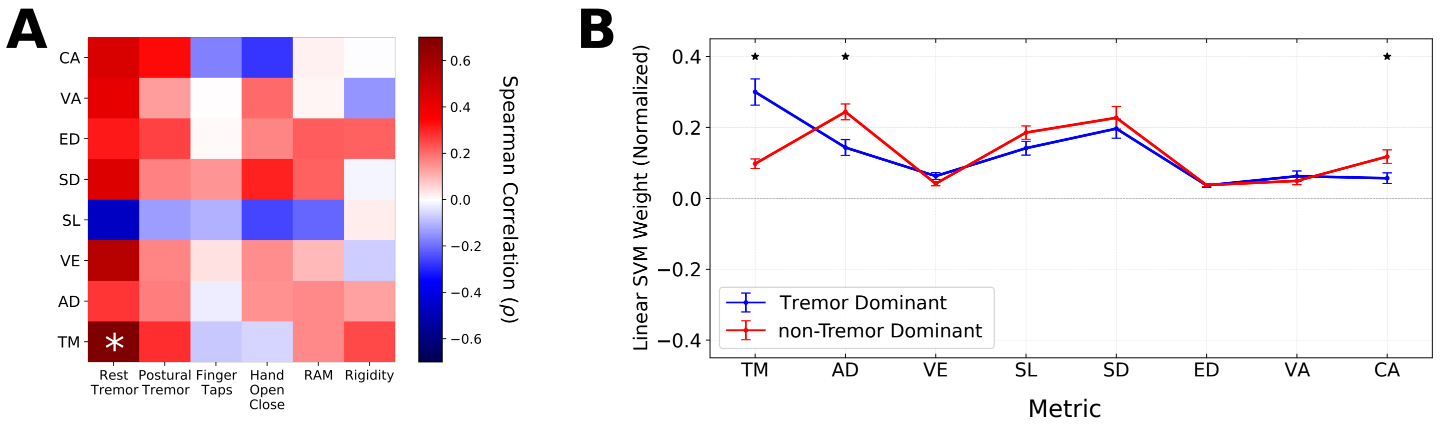

Supplementary Figure 4. *Comparison between behavioral metrics and clinical measures.* **A.** Metric distributions for each patient were calculated using 7-second bins. The median of the metric distribution was paired to the UPDRS III subscores for the upper extremity with which the patient played the task. Significant Spearman correlation was observed between tremor magnitude (TM) and Rest Tremor (*: $\rho = 0.70$, $p < 0.001$, corrected for multiple comparisons). **B.** Behavioral SVM weights were calculated separately for tremor dominant (TD) and non-tremor dominant (nTD) subtypes. TD patients (blue) exhibited significantly greater TM weights, while nTD patients (red) exhibited significantly greater absolute distance (AD) and correction angle (CA) weights (Mann-Whitney U test, corrected for multiple comparisons).

Supplementary Video. *Examples of tracking task performance by PD and control subjects.* Shown is one trial of tracking task performance by four subjects (three PD, one control). The top panels show target and cursor trajectories with a disappearing trail (not shown during actual task performance) to visualize performance more clearly. The MES is plotted in the bottom panels.
